# Hippocampal subfield associations with memory depend on stimulus modality and retrieval mode

**DOI:** 10.1093/braincomms/fcad309

**Published:** 2023-11-09

**Authors:** Etienne Aumont, Aurélie Bussy, Marc-André Bedard, Gleb Bezgin, Joseph Therriault, Melissa Savard, Jaime Fernandez Arias, Viviane Sziklas, Paolo Vitali, Nina Margherita Poltronetti, Vanessa Pallen, Emilie Thomas, Serge Gauthier, Eliane Kobayashi, Nesrine Rahmouni, Jenna Stevenson, Cecile Tissot, Mallar M Chakravarty, Pedro Rosa-Neto

**Affiliations:** NeuroQAM Research Centre, Université du Québec à Montréal (UQAM), Montreal H2X 3P2, Canada; McGill University Research Centre for Studies in Aging, McGill University, Montreal, QC H4H 1R3, Canada; Montreal Neurological Institute, McGill University, Montreal, QC H3A 2B4, Canada; Cerebral Imaging Center, Douglas Research Center, Montreal, QC H4H 1R3, Canada; Computational Brain Anatomy (CoBrALab) Laboratory, Montreal, QC H4H 1R2, Canada; NeuroQAM Research Centre, Université du Québec à Montréal (UQAM), Montreal H2X 3P2, Canada; McGill University Research Centre for Studies in Aging, McGill University, Montreal, QC H4H 1R3, Canada; Montreal Neurological Institute, McGill University, Montreal, QC H3A 2B4, Canada; Department of Neurology and Neurosurgery, McGill University, Montreal, QC H3A 1A1, Canada; McGill University Research Centre for Studies in Aging, McGill University, Montreal, QC H4H 1R3, Canada; Montreal Neurological Institute, McGill University, Montreal, QC H3A 2B4, Canada; Department of Neurology and Neurosurgery, McGill University, Montreal, QC H3A 1A1, Canada; McGill University Research Centre for Studies in Aging, McGill University, Montreal, QC H4H 1R3, Canada; Montreal Neurological Institute, McGill University, Montreal, QC H3A 2B4, Canada; Department of Neurology and Neurosurgery, McGill University, Montreal, QC H3A 1A1, Canada; McGill University Research Centre for Studies in Aging, McGill University, Montreal, QC H4H 1R3, Canada; Montreal Neurological Institute, McGill University, Montreal, QC H3A 2B4, Canada; Department of Neurology and Neurosurgery, McGill University, Montreal, QC H3A 1A1, Canada; McGill University Research Centre for Studies in Aging, McGill University, Montreal, QC H4H 1R3, Canada; Montreal Neurological Institute, McGill University, Montreal, QC H3A 2B4, Canada; Department of Neurology and Neurosurgery, McGill University, Montreal, QC H3A 1A1, Canada; Montreal Neurological Institute, McGill University, Montreal, QC H3A 2B4, Canada; McGill University Research Centre for Studies in Aging, McGill University, Montreal, QC H4H 1R3, Canada; Department of Neurology and Neurosurgery, McGill University, Montreal, QC H3A 1A1, Canada; McGill University Research Centre for Studies in Aging, McGill University, Montreal, QC H4H 1R3, Canada; McGill University Research Centre for Studies in Aging, McGill University, Montreal, QC H4H 1R3, Canada; McGill University Research Centre for Studies in Aging, McGill University, Montreal, QC H4H 1R3, Canada; McGill University Research Centre for Studies in Aging, McGill University, Montreal, QC H4H 1R3, Canada; Montreal Neurological Institute, McGill University, Montreal, QC H3A 2B4, Canada; Department of Neurology and Neurosurgery, McGill University, Montreal, QC H3A 1A1, Canada; Montreal Neurological Institute, McGill University, Montreal, QC H3A 2B4, Canada; Department of Neurology and Neurosurgery, McGill University, Montreal, QC H3A 1A1, Canada; McGill University Research Centre for Studies in Aging, McGill University, Montreal, QC H4H 1R3, Canada; Montreal Neurological Institute, McGill University, Montreal, QC H3A 2B4, Canada; Department of Neurology and Neurosurgery, McGill University, Montreal, QC H3A 1A1, Canada; McGill University Research Centre for Studies in Aging, McGill University, Montreal, QC H4H 1R3, Canada; Montreal Neurological Institute, McGill University, Montreal, QC H3A 2B4, Canada; Department of Neurology and Neurosurgery, McGill University, Montreal, QC H3A 1A1, Canada; McGill University Research Centre for Studies in Aging, McGill University, Montreal, QC H4H 1R3, Canada; Montreal Neurological Institute, McGill University, Montreal, QC H3A 2B4, Canada; Department of Neurology and Neurosurgery, McGill University, Montreal, QC H3A 1A1, Canada; Cerebral Imaging Center, Douglas Research Center, Montreal, QC H4H 1R3, Canada; Computational Brain Anatomy (CoBrALab) Laboratory, Montreal, QC H4H 1R2, Canada; Department of Psychiatry, McGill University, Montreal, QC H3A 1A1, Canada; NeuroQAM Research Centre, Université du Québec à Montréal (UQAM), Montreal H2X 3P2, Canada; McGill University Research Centre for Studies in Aging, McGill University, Montreal, QC H4H 1R3, Canada; Montreal Neurological Institute, McGill University, Montreal, QC H3A 2B4, Canada; Department of Neurology and Neurosurgery, McGill University, Montreal, QC H3A 1A1, Canada

**Keywords:** hippocampus, Alzheimer’s disease, ageing, memory, automatic segmentation

## Abstract

Hippocampal atrophy is a well-known feature of age-related memory decline, and hippocampal subfields may contribute differently to this decline. In this cross-sectional study, we investigated the associations between hippocampal subfield volumes and performance in free recall and recognition memory tasks in both verbal and visual modalities in older adults without dementia.

We collected MRIs from 97 (41 males) right-handed participants aged over 60. We segmented the right and left hippocampi into (i) dentate gyrus and cornu ammonis 4 (DG/CA4); (ii) CA2 and CA3 (CA2/CA3); (iii) CA1; (iv) strata radiatum, lacunosum and moleculare; and (v) subiculum. Memory was assessed with verbal free recall and recognition tasks, as well as visual free recall and recognition tasks. Amyloid-β and hippocampal tau positivity were assessed using [^18^F]AZD4694 and [^18^F]MK6240 PET tracers, respectively.

The verbal free recall and verbal recognition performances were positively associated with CA1 and strata radiatum, lacunosum and moleculare volumes. The verbal free recall and visual free recall were positively correlated with the right DG/CA4. The visual free recall, but not verbal free recall, was also associated with the right CA2/CA3. The visual recognition was not significantly associated with any subfield volume. Hippocampal tau positivity, but not amyloid-β positivity, was associated with reduced DG/CA4, CA2/CA3 and strata radiatum, lacunosum and moleculare volumes.

Our results suggest that memory performances are linked to specific subfields. CA1 appears to contribute to the verbal modality, irrespective of the free recall or recognition mode of retrieval. In contrast, DG/CA4 seems to be involved in the free recall mode, irrespective of verbal or visual modalities. These results are concordant with the view that DG/CA4 plays a primary role in encoding a stimulus’ distinctive attributes, and that CA2/CA3 could be instrumental in recollecting a visual memory from one of its fragments. Overall, we show that hippocampal subfield segmentation can be useful for detecting early volume changes and improve our understanding of the hippocampal subfields’ roles in memory.

## Teaser

In this study, Aumont *et al*. investigated the hippocampus, a brain region tied to memory. They found that visual memory was associated with the right hippocampus, while verbal memory was associated with both sides in older adults. Additionally, the hippocampus was smaller in the presence of local Alzheimer’s disease signature.

## Graphical Abstract

**Graphical Abstract:**
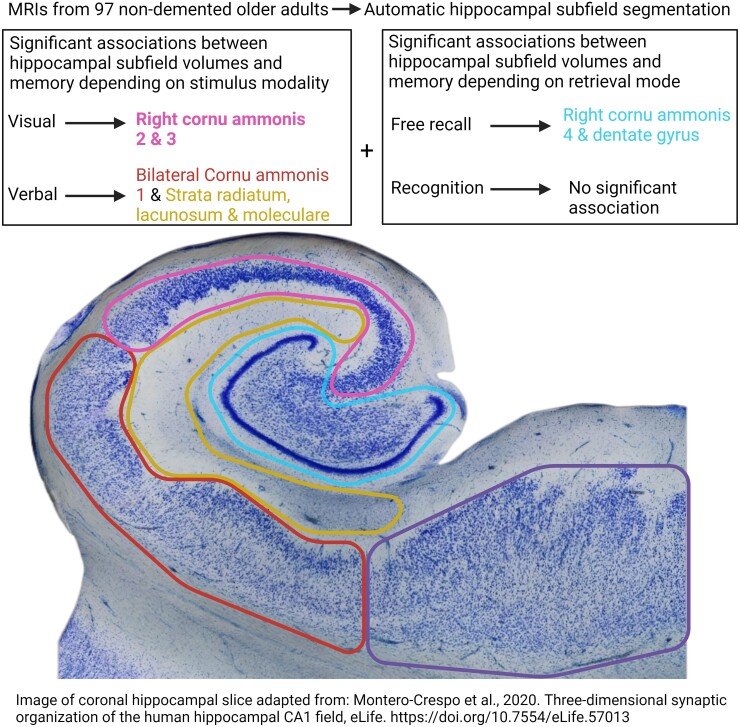

## Introduction

The hippocampus has been the focus of multiple studies on memory function and dysfunction.^1^ Hippocampal neuronal loss is observable with ageing and is detectable in presymptomatic Alzheimer’s disease, with specific subfields being more severely affected than others.^2-4^ Subdividing the hippocampus based on subfields was previously found useful in predicting Alzheimer's disease symptomatology and neuropathology severity.^5,6^ However, how changes in hippocampal subfields affect cognitively unimpaired individuals remains to be elucidated.^7^ Although some studies have found age-related volume reduction in cornu ammonis 1 (CA1) and subiculum subfields,^8,9^ others have not confirmed these findings but have instead found atrophy of the strata radiatum lacunosum and moleculare (SRLM), corresponding to the deepest layers of the CA.^10,11^ Such inconsistency might be due to different methods of subfield segmentation.^12,13^ For example, CA2, CA3 and the dentate gyrus (DG) are sometimes pooled together, while other studies consider them separately.

From a functional perspective, the hippocampus is believed to integrate and consolidate the memories’ contextual information (i.e. spatial, temporal and multimodal information).^14,15^ List learning of words may be an example of hippocampal involvement through these processes, since new contextual information is attributed to familiar stimuli.^16^ However, the level of hippocampal recruitment is expected to differ depending on the significance of the contextual information: free recall tasks, requiring richer contextual cues, are anticipated to elicit greater hippocampal involvement compared to recognition tasks.^14^ Additionally, memory acquisition of verbal and visuospatial material is thought to be processed asymmetrically between the left and right hippocampi.^17-19^ However, this distinction is inconsistent, as in several instances, verbal memory has been shown to involve both hemispheres.^20,21^ Therefore, the roles of the right and left hippocampi in verbal and visual memory processing remain unclear. One explanation may lie in the selective involvement of hippocampal subfields, rather than a contribution from the entire hippocampus. Indeed, memory acquisition of stimuli from different modalities—either visual or verbal—might depend on subfields’ structural integrity in one or both hippocampi. Moreover, as memory retrieval can be measured via free recall or recognition, it is possible that such retrieval modes might be dependent on distinct subfield circuitry.

To test this framework, the primary aim of this study was to investigate the associations between lateralized hippocampal subfield volumes and performance obtained in both verbal and visual memory tasks, measured with free recall and recognition. Based on the previous evidence described above, verbal memory measures were hypothesized to be positively correlated with subfields from both hemispheres. In contrast, the putatively more asymmetric visual memory measures were hypothesized to be positively associated with the right hemisphere subfields. In addition, recollection from free recall and from recognition was expected to be correlated with different subfields, given the hippocampal involvement in context-dependent memory. A secondary aim of this study was to investigate if the occurrence of Alzheimer's disease neuropathological changes (tau and amyloid-β accumulation) could be associated with significantly smaller hippocampal subfield volumes. Hippocampal subfield volumes were expected to be lower in pathophysiologically-laden individuals, especially for the CA1 and SRLM subfields known to be affected early in Alzheimer's disease.^22,23^

For this purpose, brain MR images of elderly individuals without dementia were segmented to extract hippocampal subfield volumes.^24^ These volumes were analysed in conjunction with performance in both verbal and visual memory tasks. To address the secondary aim, positron emission tomography (PET) imaging was performed on each participant using amyloid-β and tau radioligands.

## Materials and methods

### Participants

The cross-sectional data used in the current study originated from the Translational Biomarkers in Aging and Dementia (TRIAD) cohort. Based in Montreal, Canada, TRIAD was launched in 2017 to study Alzheimer's disease and other neurodegenerative pathologies from preclinical stages to later stages of dementia. It includes various biomarkers, genotyping, PET tracers, MRI techniques and clinical and neuropsychological assessments, ensuring optimal diagnosis.^25^ Each participants’ informed consent was obtained according to the Declaration of Helsinki, and the local ethical committee approved the protocol. Within a one-year interval, the participants had undergone the required high-resolution T_2_-weighted (T2w) MRI scan as well as the PET scans with amyloid-β tracer [^18^F]NAV4694 and tau tracer [^18^F]MK6240, in addition to a full neuropsychological assessment and *APOE* genotyping. Participants with missing data or with MRI of low quality were excluded from this study. As a result, 114 right-handed elderly participants (age range 61–84, mean: 72) either cognitively unimpaired or mild cognitively impaired were selected.

### MR imaging

All participants had an MRI using a T2w 2D turbo spin-echo sequence (TR: 14 410 ms; TE: 79 ms; FoV: 256 mm; flip angle: 120°), acquired on a 3T Siemens Magnetom Prisma scanner located at the Montreal Neurological Institute. Images were composed of 67 1-mm-thick coronal slices with a 24.8° tilt to follow the long axis of the hippocampus. Anisotropic voxels (0.7 × 0.7 × 1 mm) resulted in a high-resolution coronal image of the middle of the antero-posterior axis of the head. T2w images were preferred over T1w images for hippocampal segmentations due to their better contrast between hippocampal subfields.^26^ Additionally, a T1w MRI using Ultrafast Gradient Echo 3D sequence (TR: 2300 ms; TE: 2.96 ms; FoV: 256 mm; flip angle: 9°) with 1-mm isotropic voxels was collected. T1w images were used for initial template registration before using the T2w images for the segmentation. Quality checks for motion artefacts, as per the protocol described by Bedford *et al.*,^27^ were performed by a single rater. A rating of 1 or 2 out of a 4-point scale (1 being excellent and 4 being very poor) was required for T2w and T1w scans to be considered of good quality and be included in the study (see https://github.com/CoBrALab/documentation/wiki/Motion-Quality-Control-(QC)-Manual for examples). Motion artefacts were more salient in T2w images due to the higher resolution and later acquisition period, with a rejection rate of ∼30%.

We used the minc-bpipe-library pipeline (https://github.com/CobraLab/minc-bpipe-library) to pre-process T1w images for N4 bias field correction^28^ and to use BEaST^29^ for brain extraction and for obtaining the total intracranial volume (TIV, comprising intracranial cerebrospinal fluid, white and grey matter).

### Hippocampal segmentation

Hippocampal subfields were automatically segmented using the Multiple Automatically Generated Templates algorithm (MAGeT-Brain). Using the Winterburn and colleagues^11,30^ atlases, we identified five regions in each hemisphere, including (i) dentate gyrus and CA4 (DG/CA4); (ii) CA2 and CA3 (CA2/CA3); (iii) CA1; (iv) SRLM; and (v) subiculum. These atlases were defined based on five ultra-high-resolution MRIs from which both hippocampi were manually segmented into the five regions mentioned above. A modified version of the protocol published by Pipitone *et al.*^31^ allowed the segmentation of the higher-resolution T2w slabs using the corresponding whole-brain T1w images to facilitate the segmentation.^7,10^ When used on the T1w images alone with the original protocol, the Dice’s Similarity Coefficients with manual segmentation were previously found to be between 0.55 and 0.65 in CA1, DG/CA4 and subiculum subfields.^31^

Hippocampal subfield segmentation was performed in two steps. First, to select the best template brains, MAGeT-Brain coregistered the five atlas brains to each participant’s image and applied the same transformation on their corresponding subfield segmentation. This allowed for each voxel to be labelled. This labelling generated five hippocampal segmentations—one per atlas—for a given T2w image. These five segmentations were unified into a single segmentation for each participant using a majority vote approach. A single rater then manually inspected each participant’s segmentation to select 21 of the best segmented brains to be used as templates. These 21 templates were chosen based on a balance between high segmentation quality and good participant representativity with the sample demographic features.

**Figure 1:**
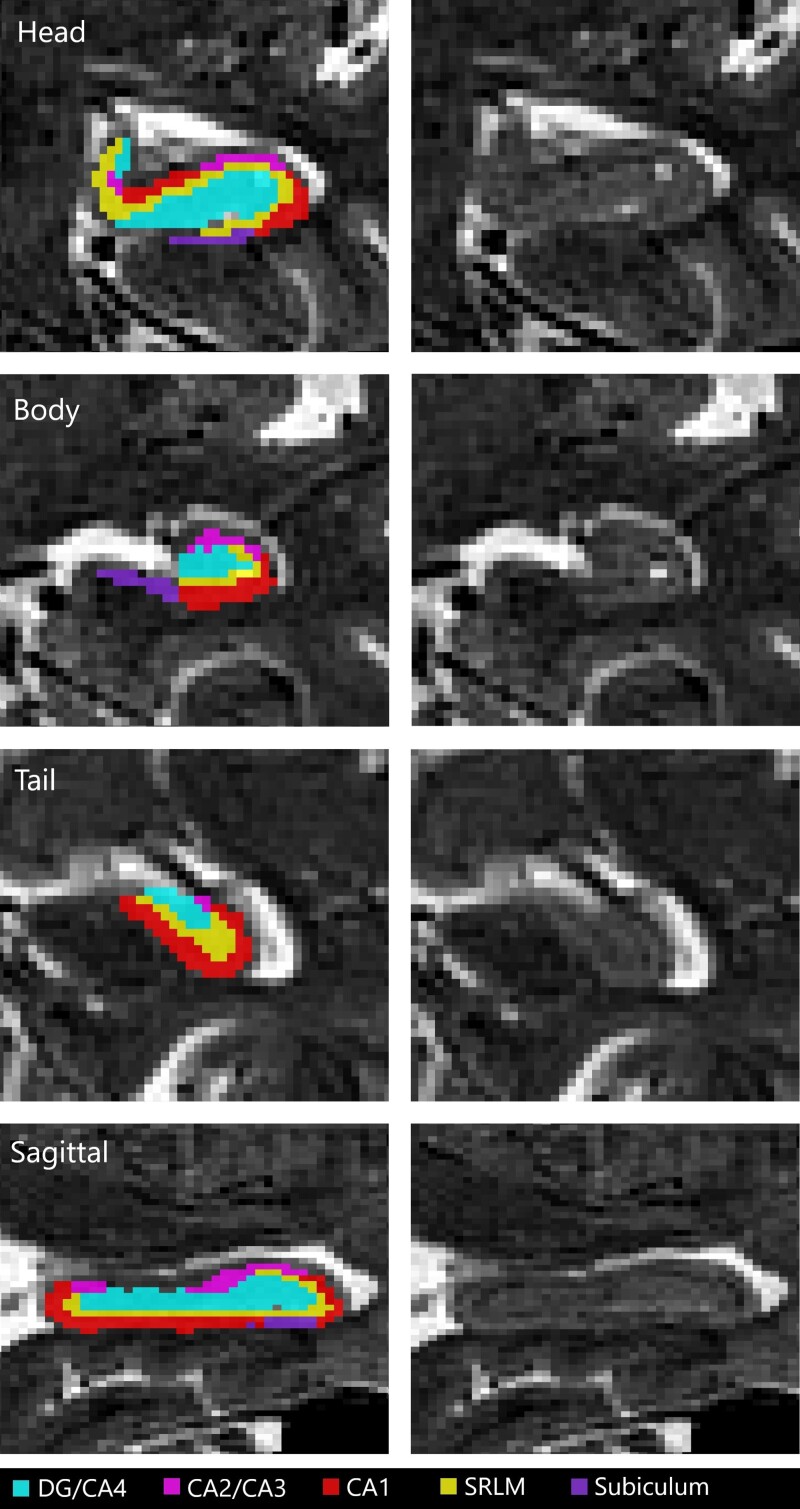
**An example of hippocampal segmentation resulting from the MAGeT-Brain algorithm over a T2-weighted MRI from a TRIAD subject.** A sagittal slice and coronal slices of the hippocampus head, body and tail are displayed with and without a subfield mask overlay. CA, cornu ammonis; DG, dentate gyrus; SRLM, strata radiatum, lacunosum and moleculare.

In the second step, the 21 selected brains were used as templates—each with five segmentations defined from the atlases—were coregistered to each participant’s image, producing 105 segmentations per participant (5 atlases × 21 templates). These 105 segmentations were combined to obtain one final unified segmentation per participant through a majority vote (Fig. 1). A single rater assessed the unified segmentations for quality control, and hippocampal volumes were extracted from the labels for later analyses. Before any statistical analyses, all hippocampal volumes were adjusted for TIV through a linear model regression. TIV is an essential covariate when investigating brain structure volumes, as a smaller hippocampal volume might stem from a smaller TIV instead of a specific atrophy.

### Amyloid-β and tau-PET imaging

All participants had a brain PET scan with [^18^F]MK-6240, a tracer with adequate specificity for tau neurofibrillary tangles.^32,33^ They also had amyloid-β plaques imaging performed with the [^18^F]AZD4694 (otherwise known as [^18^F]NAV4694) tracer. PET data were acquired with a Siemens High Resolution Research Tomograph (HRRT) (point-spread function of 2.4-mm full-width half-maximum). [^18^F]MK-6240 images were acquired 90–110 minutes post-injection, and the [^18^F]AZD4694 images were acquired 40–70 minutes post-injection. Images were reconstructed with the ordered subset expectation maximization algorithm on a four-dimensional volume with 300-second frames (four frames for [^18^F]MK-6240 and six frames for [^18^F]AZD4694).^34^ Following each PET scan, a 6-minute transmission scan was conducted with a rotating point of ^137^Cs source for attenuation correction. Images underwent corrections for dead time, decay, random and scattered coincidences.

We obtained PET image transformation matrices from linear PET registration to the bias field corrected T1w image space. In parallel, T1w images were linearly and non-linearly registered to the ADNI template space. These transformations were concatenated and applied to the PET image using Advanced Normalization Tools.^35^ PET images were spatially smoothed using an 8-mm full-width half-maximum Gaussian kernel. Standardized uptake value ratio (SUVR) maps were generated using the inferior cerebellar grey matter as a reference region for [^18^F]MK6240 images and the whole cerebellar grey matter as a reference region for [^18^F]AZD4694 scans.^34,36^ We obtained global amyloid-PET signal on average SUVRs from the precuneus, prefrontal, orbitofrontal, parietal, temporal, anterior and posterior cingulate cortices.^37^ Participants were categorized as either amyloid-β positive (A+) or negative (A−) based on a threshold that has been validated specifically for [^18^F]AZD4694 scans using data from the same cohort (SUVR > 1.55).^38,39^ We established hippocampal tau positivity (T+) or negativity (T−) based on the average SUVR in the Braak stage II area (Supplementary Fig. 1), surpassing a threshold established in a previous TRIAD study (SUVR > 0.9940).^40^ We chose Braak stage II as it is mostly contained within the hippocampus while attained at an early stage, with abnormalities preceding cognitive decline.^40^ This region of interest (ROI) is mostly made up of the hippocampus body and the posterior half of the head, with a smaller cluster situated within the entorhinal cortex.

### Montreal Cognitive Assessment

General cognitive status was assessed using the Montreal Cognitive Assessment (MoCA), a well-validated and widely used cognitive test for the screening of dementia and cognitive impairment (range 0–30, with 30 being a perfect score).^41^ One point was added to the scores of participants with <13 years of education, as suggested by the authors of this scale.^41^

### Memory assessment

Memory was assessed using Rey Auditory Verbal Learning Test (RAVLT) and Aggie Figures Learning Test (AFLT) scores. The RAVLT consists of reading aloud a series of 15 commonly used words (list A) at a rate of one word per second. Immediately after the list has been read, participants are requested to repeat as many words as they can remember. Five trials are performed using the same words in the same order. This is followed by a list B trial, comprising different words, followed by a trial where the participants attempt to produce list A without it being read beforehand. Thirty minutes later, participants are asked to recollect list A (delayed free recall).^42^ Immediately after this, a delayed recognition trial (RAVLT-R) is administered, presenting a list of 50 words orally, one by one, to the participant, who must judge whether or not they were in list A. Except for the delayed recognition trial, the AFLT follows the same structure through the visual presentation of simple abstract figures, with participants being asked to draw their answers.^43^ The visual recognition trial (AFLT-R) consists of 50 figures presented one by one, with the subjects being asked to classify items as either from one of the previous series or from a distractor series (items that had never been presented before). The images used in the AFLT are designed not to be easily verbalized, so mental images are the most efficient way of memorizing them. Due to their unique nature, they are not expected to be familiar to participants before completing the task.

Delayed free recall performance was defined as the number of words or figures successfully recalled after a 30-minute delay (ranging from 0–15, with 15 being a perfect recall), also known as trial 7 (RAVLT7 and AFLT7). To account for both false recognitions (distractors) and correct recognitions during recognition trials, we calculated the discrimination index (dʹ) between the learned and distractor lists according to the signal detection theory.^44^ In the case of the AFLT-R, we calculated dʹ for series A and B separately. However, only the dʹ for list A could be calculated for the RAVLT-R. A negative dʹ indicated a higher detection rate for the distractors than for the learned stimuli. These instances were cases where no learning had occurred and were therefore not included in the analyses. Based on average scores from trials 1 through 7, both RAVLT and AFLT displayed similar learning rates, suggesting that similar levels of encoding and consolidation were achieved in both modalities.

### Statistical analyses

We conducted all statistical analyses with RStudio, version 2021.9.1.372,^45^ using two-tailed *P* = 0.05 as a statistical significance threshold. As a measure of correction for multiple comparisons, we adjusted the *P*-values using the Benjamini–Hochberg false discovery rate (FDR) procedure for each analysis group.^46^ Those corrected *P*-values are reported in the results as *P*_corr_. To improve the correspondence of data visualization to the statistical analyses, adjusted values, which were obtained by adding the mean to the residuals from covariate regressions, are depicted in all the figures.

Semi-partial regressions were performed associating RAVLT7 and AFLT7 scores with each hippocampal volume, using age, years of education and sex as covariates, and TIV was used as a specific covariate for subfield volumes. We included demographic covariates because of the differential impact that age, sex and years of education might have on neuropsychological test performance and the levels of brain atrophy. An FDR procedure was performed on all 20 results from these analyses. In addition, we performed semi-partial regressions associating three recognition measures with hippocampal volumes: RAVLT-R dʹ, AFLT-R series A dʹ and AFLT-R series B dʹ using age, years of education and sex as covariates. FDR was applied independently for results from each three recognition measures because they were obtained from distinct protocols. The asymmetry of associations was assessed with a paired *t*-test comparing left and right standardized *β* estimates on each set of analyses.

We performed two sets of ANCOVAs, comparing the hippocampal subfield volumes of (i) A+ with A− and (ii) T+ with T− individuals. Since both categorizations are largely overlapping, a single FDR procedure was used on all 20 results to limit type 1 error inflation. We used age, years of education, presence of *APOE ε4* and TIV as covariates. We verified normality using the Shapiro test, homogeneity of variance using *F*_max_ ratios and multicollinearity using variance inflation factor. Adjusted standardized mean differences (SMD) were used as measures of effect sizes. Confidence intervals of 95% for all effect size measures can be found in Supplementary material. Group comparisons on continuous dependent demographic and cognitive variables (age, years of education, MoCA, TIV, RAVLT and AFLT) were conducted using *t*-tests to verify group differences. Comparisons on categorical variables (*APOE ε4*, sex) were conducted using chi-squared tests.

Based on a previous study, we anticipated that the standardized *β*-values of partial regressions between memory scores and subfield volumes would be ∼0.35.^47^ Given an alpha of 0.01 (to account for FDR correction), a sample size of 85 was determined to achieve a statistical power of 80%.

## Results

### Demographic data

After proceeding with hippocampal segmentation, we excluded the participants’ data extracted from poor quality segmentations from our database, with left and right volume quality being treated independently. This resulted in a total of 97 individuals with either or both hippocampal segmentation data available (left: *n* = 77, right: *n* = 89). Among the 97-participant sample, we identified 35 *APOE ε4* carriers, 30 A+ and 34T+ individuals. There were 54 A−T−, 13 A−T+, 9 A+T− and 21 A+T+. See Table 1 for a demographic and cognitive data summary.

**Table 1:** Demographic and cognition table

	Average (standard deviation) [min, max]	Amyloid-based categorization			Tau-based categorization		
		A+	A−	*P*	T+	T−	*P*
*n*	97	30	67	NA	34	63	NA
Age	71.8 (5.0) [61, 84]	73.3 (4.7) [65, 84]	71.1 (5.0) [61, 82]	0.039*	73.2 (4.5) [65, 82]	71.0 (5.1) [61, 84]	0.031*
Education (years)	15.4 (3.8) [6, 26]	15.3 (3.8) [6, 25]	15.4 (3.8) [7, 26]	0.935	15.4 (4.0) [7, 26]	15.4 (3.8) [6, 25]	0.999
Sex (female)	56 (57.7%)	18 (60%)	38 (56.7%)	0.936	18 (52.9%)	38 (60.3%)	0.627
APOE ε4 carriers	35 (36.1%)	14 (46.7%)	21 (31.3%)	0.221	18 (52.9%)	17 (27%)	0.020*
MoCA	27.1 (2.3) [19, 30]	25.9 (2.8) [19, 30]	27.6 (1.9) [22, 30]	0.003*	26.3 (2.6) [19, 30]	27.5 (2.1) [21, 30]	0.021*
Number of MCI	26 (26.8%)	18 (60%)	8 (11.9%)	0.000*	16 (47.1%)	10 (15.9%)	0.002*
RAVLT7	9.73 (3.73) [0, 15]	7.30 (4.01) [0, 14]	10.8 (3.05) [0, 15]	0.000*	8.53 (4.08) [0, 15]	10.4 (3.39) [0, 15]	0.028*
AFLT7	9.67 (3.76) [0, 15]	8.00 (3.59) [0, 14]	10.4 (3.62) [0, 15]	0.003*	8.76 (4.02) [0, 15]	10.2 (3.56) [0, 15]	0.095
TIV (cm³)	1350 (124) [1078, 1698]	1369 (133) [1097, 1660]	1340 (120) [1078, 1698]	0.306	1361 (129) [1097, 1660]	1342 (122) [1078, 1698]	0.468

^*^Significant at *P* < 0.05.

### Hippocampal subfield volumes and memory performance

**Figure 2:**
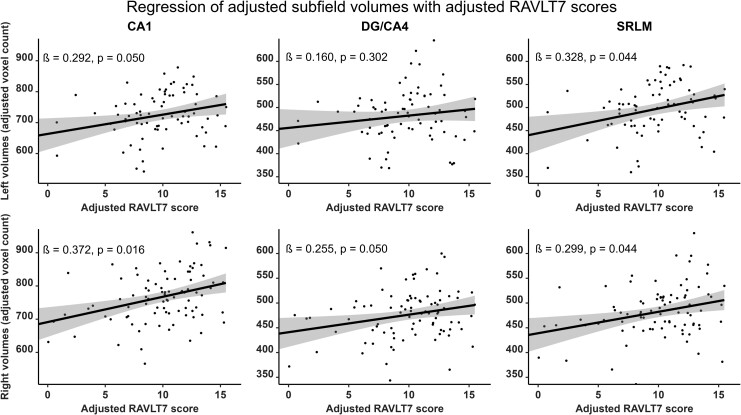
**Representation of semi-partial regressions between hippocampal subfield volumes and delayed verbal free recall performance (RAVLT7).** Data were adjusted for age, sex, years of education and total intracranial volume. Left subfield *n* = 77, right subfield *n* = 89. Displayed *P*-values are FDR-corrected. *β*, standardized semi-partial regression coefficient; CA, cornu ammonis; DG, dentate gyrus; SRLM, strata radiatum, lacunosum and moleculare.

**Figure 3:**
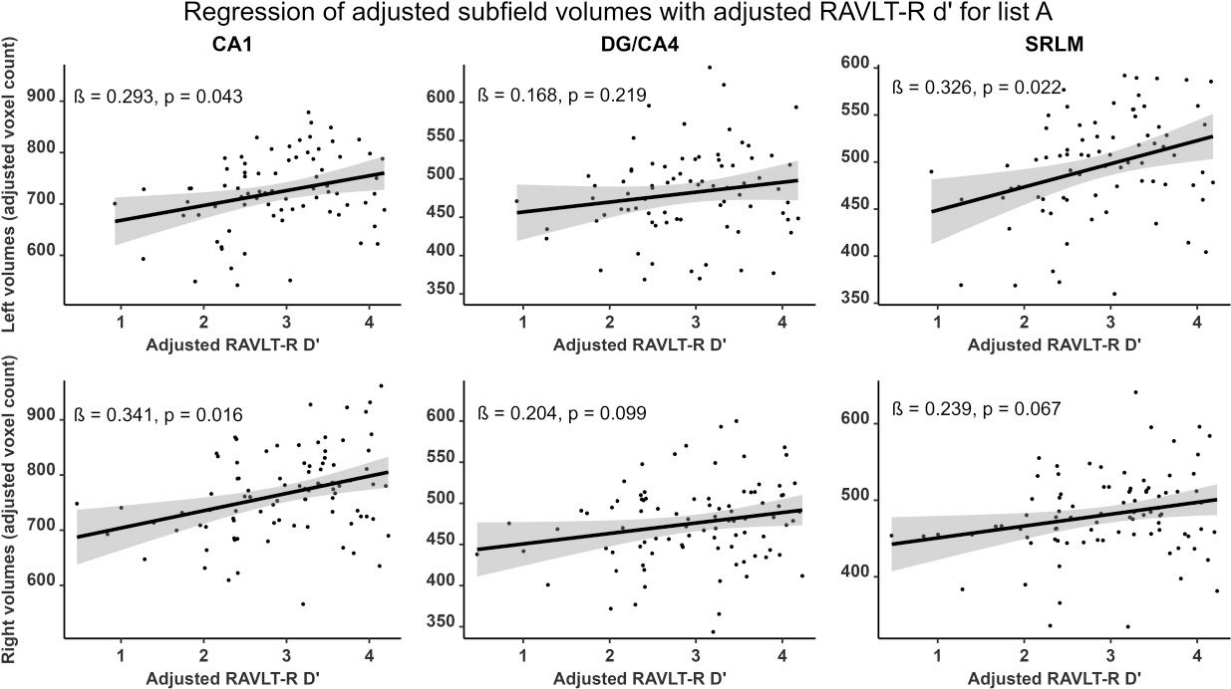
**Representation of semi-partial regressions between hippocampal subfield volumes and delayed verbal recognition (RAVLT-R) discrimination index (d′).** Data were adjusted for age, sex, years of education and total intracranial volume. Left subfield *n* = 77, right subfield *n* = 89. Displayed *P*-values are FDR-corrected. *β*, standardized semi-partial regression coefficient; CA, cornu ammonis; DG, dentate gyrus; SRLM, strata radiatum, lacunosum and moleculare.

**Figure 4:**
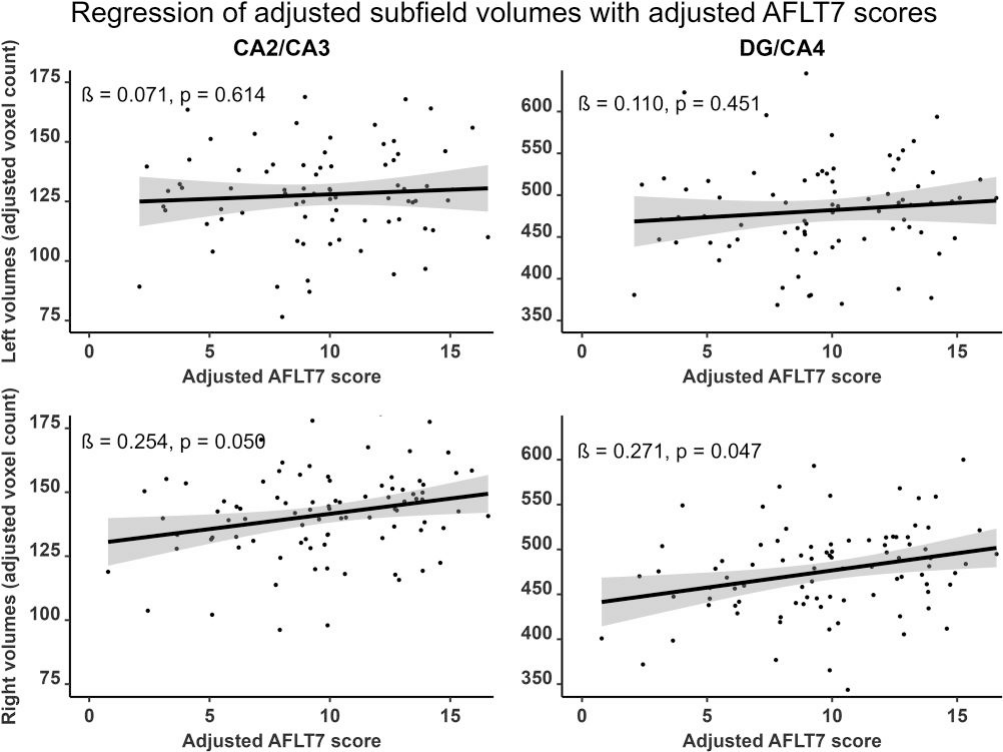
**Representation of semi-partial regressions between hippocampal subfield volumes and delayed visual free recall performance (AFLT7).** Data were adjusted for age, sex, years of education and total intracranial volume. Left subfield *n* = 77, right subfield *n* = 89. Displayed *P*-values are FDR-corrected. *β*, standardized semi-partial regression coefficient; CA, cornu ammonis; DG, dentate gyrus.

Verbal and visual modalities, as well as free recall and recognition, were differently associated with hippocampal subfields. RAVLT7 was significantly and positively correlated with the bilateral CA1 (right: *β* = 0.372; *P*_corr_ = 0.016; left: *β* = 0.292; *P*_corr_ = 0.050), the bilateral SRLM (right: *β* = 0.299; *P*_corr_ = 0.044; left: *β* = 0.328; *P*_corr_ = 0.044) and the right DG/CA4 (*β* = 0.255; *P*_corr_ = 0.050) subfield volumes (Fig. 2, Supplementary Fig. 2 and Supplementary Table 1). RAVLT-R dʹ was positively correlated with the bilateral CA1 (right: *β* = 0.341; *P*_corr_ = 0.016; left: *β* = 0.293; *P*_corr_ = 0.043) as well as the left SRLM (*β* = 0.326; *P*_corr_ = 0.022) subfield volumes (Fig. 3, Supplementary Fig. 3 and supplementary Table 2). In contrast, the AFLT7 scores significantly and positively correlated with the right DG/CA4 (*β* = 0.271; *P*_corr_ = 0.047) and the right CA2/CA3 (*β* = 0.254; *P*_corr_ = 0.050) subfield volumes (Fig. 4, Supplementary Fig. 4 and Supplementary Table 3). Neither AFLT-R dʹ for series A nor series B were significantly correlated with subfield volumes (Supplementary Tables 4 and 5, Supplementary Figs 5 and 6).

**Figure 5:**
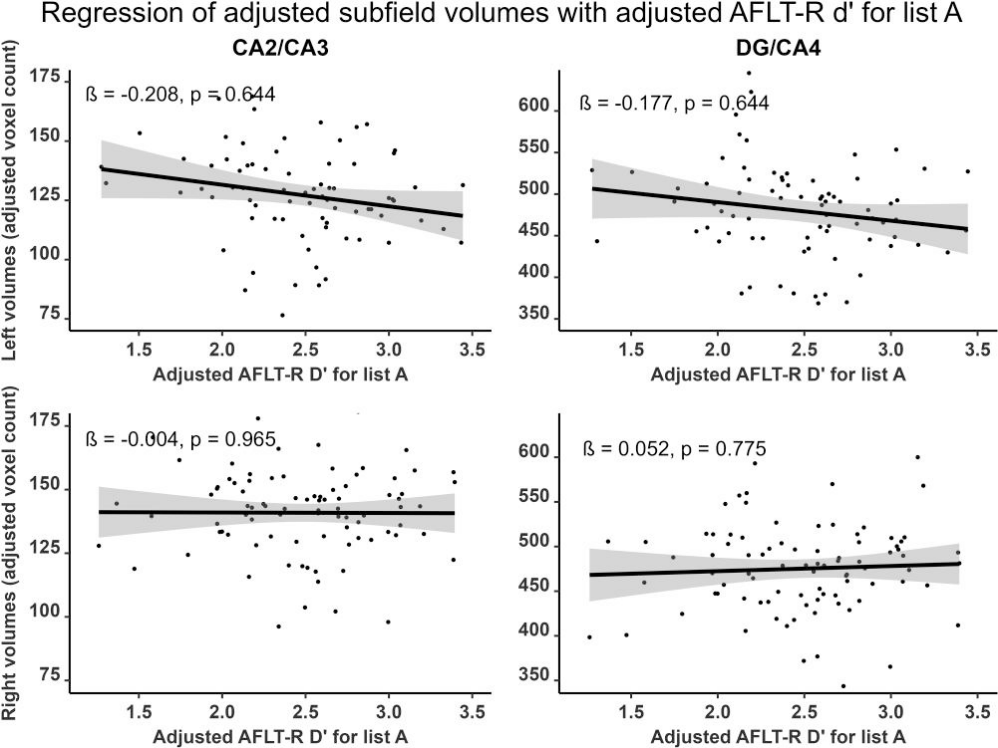
**Representation of semi-partial regressions between hippocampal subfield volumes and delayed visual recognition (AFLT-R) discrimination index for series A (d′).** Data were adjusted for age, sex, years of education and total intracranial volume. Left subfield *n* = 77, right subfield *n* = 89. Displayed *P*-values are FDR-corrected. *β*, standardized semi-partial regression coefficient; CA, cornu ammonis; DG, dentate gyrus.

To verify the asymmetry of these associations, we compared the regression sizes between memory performance and subfield volumes between each hemisphere. We found no asymmetry of the regressions between subfield volumes and either RAVLT7 scores (Left mean *β* = 0.230; Right mean *β* = 0.244; *t*(8) = 0.246, *P* = 0.812) and RAVLT-R dʹ (Left mean *β* = 0.232; Right mean *β* = 0.194; *t*(8) = 1.091, *P* = 0.337). In contrast, regressions of AFLT scores with subfield volumes were significantly stronger in the right hemisphere with the AFLT7 (Left mean *β* = 0.075; Right mean *β* = 0.204; *t*(8) = 2.865, *P* = 0.021) and the AFLT-R series A dʹ (Left mean *β* = −0.124; Right mean *β* = 0.062; *t*(8) = 4.854, *P* = 0.001). The latter was particularly distinct from other results due to trends towards negative regressions with the left hemisphere driven by the left CA2/CA3 (*β* = −0.208) and the left DG/CA4 (*β* = −0.177) subfield volumes (Fig. 5 and Supplementary Fig. 5). Regressions with the series B dʹ, were neither negative nor asymmetric (Left mean *β* = −0.015; Right mean *β* = 0.040; *t*(8) = 1.084, *P* = 0.310).

### Amyloid-β and tau group comparisons

We did not find any significant difference between A+ and A− participants in terms of TIV, years of education, sex and *APOE ε4* carrying frequency. A+ participants were significantly older than A− participants, with poorer cognition scores on the MoCA, RAVLT7 and AFLT7 scores (see Table 1). We did not find significant subfield volume differences between A+ and A− participants (Supplementary Table 6 and Supplementary Fig. 7).

**Figure 6:**
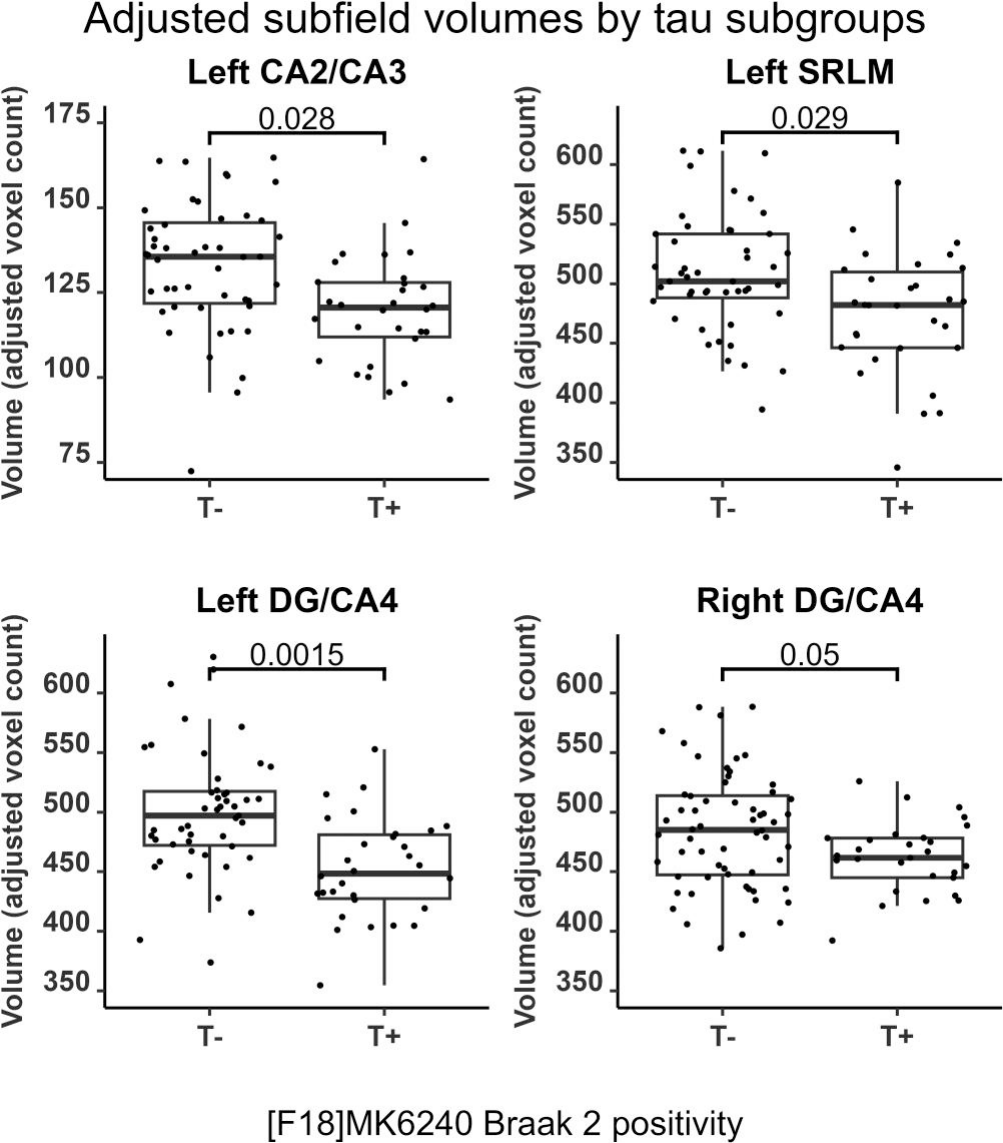
**Hippocampal subfield volumes for tau-positive (T+) and tau-negative (T−) participants.** Data points are adjusted for age, sex, total intracranial volume, years of education and APOE ε4 carrier status. Displayed *P*-values represent results from FDR-corrected ANCOVAs. CA, cornu ammonis; DG, dentate gyrus; SRLM, strata radiatum, lacunosum and moleculare.

No significant difference for TIV, years of education, sex and scores on the AFLT7 was found between T+ and T− participants. T+ participants were more likely to be *APOE ε4* carriers, tended to be older and had poorer cognitive scores on the MoCA and RAVLT7 (see Table 1). When compared to T− participants, the T+ participants displayed significant atrophy of the left SRLM (adjusted SMD = 0.649; *P*_corr_ = 0.029), the bilateral DG/CA4 (right: adjusted SMD = 0.552; *P*_corr_ = 0.050; left: adjusted SMD = 0.876; *P*_corr_ = 0.002) and the left CA2/CA3 (adjusted SMD = 0.686; *P*_corr_ = 0.028) subfields (Fig. 6). Trends for T+ atrophy when compared to T− participants in the right SRLM (adjusted SMD = 0.538; *P*_corr_ = 0.059) and the bilateral CA1 (right: adjusted SMD = 0.585; *P*_corr_ = 0.056; left: adjusted SMD = 0.491; *P*_corr_ = 0.086) subfields did not survive FDR correction (Supplementary Table 7 and Supplementary Fig. 8).

A qualitative investigation of combined amyloid and tau classification criteria revealed that, for 9 out of 10 subfields, volumes in the A−T+ group were lower than in the A+T−, and they were comparable to the A+T+ group. By contrast, the A+T− group showed volumes closer to the A−T− group (Supplementary Fig. 9).

## Discussion

In the present study, we examined the relationships between hippocampal subfield volumes and memory performance in elderly individuals. We also described the subfield volume differences in cognitively normal older adults presenting with and without significant Alzheimer's disease pathology. Verbal material retrieved either from free recall or recognition was significantly associated with bilateral hippocampal volumes of the CA1 and SRLM. Free recall for either verbal or visual material was associated with the right DG/CA4 volume. Moreover, visual material tended to be preferentially associated with CA2/CA3. We did not find significant specific subfield associations with recognition common to both verbal and visual material. We also found significantly smaller bilateral DG/CA4, left SRLM and left CA2/CA3 volumes in T+ individuals compared to T− participants, but differences in A+ individuals compared to the A− group were non-significant.

### Specific subfield associations with memory are consistent with their putative function

The association of CA1 and SRLM with verbal memory, irrespective of the free recall or recognition processes, is concordant with the literature showing that CA1, including its deeper strata, encompassing most of the SRLM subfield, is associated with recollection.^48^ Radhakrishnan *et al.*^49^ have reported similar findings, where the RAVLT delayed recollection performance most strongly correlated with bilateral CA1 subfield integrity. Traditionally, verbal memory tasks were associated with the left hippocampus, although it is now generally accepted that both hippocampi are involved in verbal memory tasks.^16,20^ Therefore, the bilateral involvement found here is concordant with this contemporary view. However, it may also indicate that verbal modality is not the only feature to consider in these results.^19,50^ Indeed, bilaterality may be involved here because the verbal stimuli could have been recollected by using both verbal and non-verbal strategies. As suggested by others,^51^ the RAVLT stimuli may be recalled by visualizing images associated with the words (e.g. drum, river and curtain) instead of the verbal component *per se*. With such an alternative recollection strategy, CA1 and SRLM subfields from both hemispheres may contribute to the recall without contradicting the long-standing theory of verbal memory lateralized to the left hippocampus.

Our results also show that free recall was associated with the right DG/CA4 subfield volumes, whether it involves visual or verbal material. This is consistent with previous results obtained in patients with mild cognitive impairment showing a significant relationship between the DG volume and the RAVLT performance in the immediate and delayed free recall.^52^ Many studies have suggested that the DG is involved in stimulus pattern separation during learning, an essential process allowing similar memories to be encoded distinctly from one another through specific neural representation.^53,54^ This function may be essential for indexing the representations, thus allowing for an efficient recall based on distinctive characteristics. Such an index of representations would improve the mental search for the learned stimuli, regardless of their modality. According to Cowell *et al.*s’^14^ framework, mental search or indexing is not required during the recognition process. This may explain why DG/CA4 was not significantly associated with recognition performance.

In contrast with the verbal free recall and recognition retrieval modes, the visual free recall was particularly associated with right hippocampal subfields, including the DG/CA4 and CA2/CA3. While DG/CA4 was a non-specific predictor of both verbal and visual free recall scores, the CA2/CA3 subfield was only associated with visual free recall. We speculate that the association of CA2/CA3 with the visual free recall is due to the ability of CA3 to perform pattern completion: the reconstruction of a whole memory based on its fragments.^55^ Using this process, one may recollect a visual figure’s complete memory by recalling a visual fragment or a verbal description that is associated to it. It may also be essential in recollecting a series of figures based on similar characteristics, allowing for better visual stimulus recollection performance. There are several accounts of visual memory being associated with the right hippocampus.^16,43^ We take this idea one step further by suggesting that the CA3 is intrinsically more closely tied to visual recall strategies. This may be related to the fact that visual stimuli are easily identifiable through visual fragments or descriptions.

### Subfield associations with memory may reflect differential circuit involvement

Two hippocampal circuits are generally assumed to be involved in different memory processes. On the one hand, the trisynaptic pathway is an entorhinal–hippocampal loop of information processing found in mammals.^56,57^ Its first step involves the perforant path, where the DG and CA3 receive fibres from the transentorhinal and entorhinal cortices. In the second step, mossy fibres connect the DG to the CA3. As a third step, Schaffer collaterals from the DG and CA3 synapse at CA1. In the last step, neurons from CA1 then send back synapses to the entorhinal cortex.^56,57^ This circuit is considered instrumental in generating a contextual representation to enrich memory traces. This added information can serve as cues for the recollection in a similar context, increasing the efficacy of search strategies during recall.^58^ In this respect, the trisynaptic circuit is of primary importance during free recalls. This may explain the association observed here between DG/CA4, CA2/CA3, CA1 and SRLM with free recall performance.

The associations of CA1 and SRLM with verbal recognition might be explained by another hippocampal circuit: the temporoammonic pathway. The latter is made of fibres from the entorhinal cortex layer III reaching the SRLM of CA1. This pathway is thought to be crucial during recognition by matching the perceived stimulus and its context with a previous representation of that stimulus in the same context.^59,60^ This pathway would allow the hippocampus to detect contextual novelty, which corresponds to the unfamiliar conjunction of a familiar stimulus and its context.^15^ In the case of RAVLT-R, participants had to recognize putatively known words as having been associated or not with a learned word list. This requires the contextual novelty detection system, hence explaining the association between RAVLT-R performance and CA1 and SRLM volumes.^14^

One can argue whether the two hippocampal circuits described above are essential for the completion of the AFLT-R. Indeed, the AFLT consists of abstract figures that were never seen before. These figures may therefore be recognized from unfamiliarity alone. Familiarity corresponds to the search of the stimulus that best matches among previously recorded representations.^14^ If none is found, the stimulus is considered unfamiliar. This familiarity acts as a stimulus novelty detector, as opposed to the contextual novelty detection enabled by the hippocampus. This process involves the perirhinal cortex and may render the hippocampal contextual novelty detection unnecessary during a recognition task involving stimuli that were never encountered before the experiment.^61^ The perirhinal cortex-dependent stimulus novelty detector may explain the negligible association between hippocampal subfields and AFLT-R performance.

### Hippocampal tau pathology is associated with hippocampal atrophy

Amyloid-β-based comparisons showed no significant atrophy of subfield volumes in A+ individuals compared to the A− group. In contrast, tau-based comparisons showed significant atrophy of the bilateral DG/CA4, left CA2/CA3 and left SRLM in T+ individuals. Marizzoni *et al.*^62^ have previously found strong associations between the volume of DG/CA4 and combined amyloid-β and phosphorylated tau CSF biomarkers positivity. Therefore, our results reinforce the idea that, rather than being a consequence of both amyloid-β and tau neuropathologies, hippocampal atrophy may be caused primarily by tau-associated toxicity. Although hippocampal atrophy has previously been associated with amyloid-β plaque accumulation, this is now controversial as hippocampal atrophy and amyloid-β accumulation are increasingly considered distinct co-occurring events,^63^ and because several studies have linked amyloid-β accumulation with subsequent tau accumulation. Tau protein accumulations have been seen as a likely cause for hippocampal atrophy, with early tau pathology appearing specifically in the medial temporal lobe (MTL), diffusing throughout the hippocampus early in the disease process, starting from strata radiatum and oriens of CA1.^22,23^ Moreover, tau progression is associated with memory decline, a prime clinical indicator of Alzheimer's disease progression.^64,65^ This association is thought to be mediated by the atrophy of specific MTL structures while downstream of amyloid-β pathological processes.^66,67^

### Study limitations

One may remark that the presence of subjects with mild cognitive impairment and Alzheimer's disease neuropathological changes may represent a weakness to this study. However, our sample had Alzheimer's disease neuropathological features quantitatively similar to those reported in a recent meta-analysis performed in healthy normal subjects.^68^ The wide range of cognitive performances and brain aging changes enhances the statistical power when investigating the biological substrate of cognitive function.^69,70^ Therefore, excluding participants with tauopathy and amyloidosis would likely reduce the association between volumes and cognition while rendering the sample less representative of the normal elderly population Still, to avoid specifically measuring the effect of Alzheimer's disease, subjects with dementia were excluded from the study.

Another potential limitation of the current study is that RAVLT and AFTL are not perfect analogues to one another: the recognition protocols were slightly different. Therefore, differences between both tests could not be exclusively attributed to the verbal or visual modality. With that in mind, we strove to transcend this limitation through this discussion. Longitudinal studies would also be useful to validate our findings to (i) limit inter-individual biases; (ii) assess the predictive value of Alzheimer's disease pathology progression within hippocampal subfields; and (iii) assess the predictive value of hippocampal subfields as a function of memory changes. Additionally, future studies should focus on disentangling memory processes, such as learning, storage or recall, to allow further characterization of the role of the hippocampal subfields in memory.

We must also mention that our volume estimates are highly dependent on the performance of the MAGeT segmentation protocol.^24,31^ Many segmentation techniques and atlases exist, and none are exempt from biases. Indeed, MAGeT uses manual segmentations of hippocampal subfields that were based on validated post-mortem histological criteria, estimating actual structure boundaries through practical, although imperfect, geometrical criteria.^71^ This segmentation protocol is able to suitably extract the hippocampal subfield volumes even from lower quality images such as from standard 1-mm isotropic voxel T1w MRI.^31^ Therefore, MAGeT is one of the most effective subfield segmentation method available at the time.

Recent studies suggest that hippocampal subfields may not be distinct functional units. For instance, Chang *et al.*^72^ report that the functional organization within the hippocampus is more intricate than what lamellar anatomical divisions might capture. For one, the posterior and anterior hippocampus may be biased towards pattern separation and completion, respectively.^73^ In addition, we did not statistically compare association strengths with memory measures. This means that we cannot establish that a specific subfield is more strongly associated with one type of memory than another. We therefore encourage caution when attributing—and not attributing—specific cognitive roles to hippocampal subfields.

## Conclusion

In the present cross-sectional study, we have found specific patterns of hippocampal subfields associated with the verbal or visual modalities, as well as with the retrieval mode by free recall or recognition. Verbal memory, as assessed with the RAVLT, was associated with bilateral hippocampal CA1 and SRLM volumes, irrespective of the retrieval mode. The free verbal and visual material recall was associated with the right DG/CA4 subfield. In addition, free recall of visual material in the AFLT was significantly associated with the right CA2/CA3 subfields. Visual recognition in the AFLT was not significantly associated with any hippocampal subfield. These results support the framework proposed based on lesion and animal studies for the role of medial temporal lobe structures in memory. Importantly, we have identified several hippocampal subfields with a significant volume reduction in elderly individuals without dementia presenting with a significant hippocampal tau load. The strategy of using hippocampal subfields to assess hippocampal functioning in memory performances might bridge the gap between neurophysiology and the cognitive sciences.

## Supplementary Material

fcad309_Supplementary_Data

## Data Availability

Anonymized data will be shared upon request to the study’s senior author from a qualified academic investigator for replicating the procedures and results presented in this article. Any data and materials that can be shared will be released via a material transfer agreement. Data are not publicly available due to information that could compromise the privacy of research participants, and the script used for data analyses may be found among the Supplementary material. Related documents, including study protocols and informed consent forms, can also be made available upon request (https://triad.tnl-mcgill.com/contact-us/).
