# Supplementary material for: Hippocampal subfield associations with memory depend on stimulus modality and retrieval mode
Source: Brain Commun. 2023 Nov 9;5(6):fcad309. doi: 10.1093/braincomms/fcad309 (PMC10681971; doi:10.1093/braincomms/fcad309)
Supplement: fcad309_Supplementary_Data [file fcad309_supplementary_data.docx]

**Supplementary materials**

Supplementary table 1: Summary of statistical tests for linear models of hippocampal subfield volumes with RAVLT delayed recall performance adjusted for age, sex, total intracranial volume and years of education.

| Hippocampal subfield | Hemisphere | DF | Uncorrected p-value | Adjusted p-value | Estimated partial β [95% CI] |
| --- | --- | --- | --- | --- | --- |
| CA1 | Left | 72 | .017 | .050* | .292 [.053; .530] |
|  | Right | 84 | .001 | .016* | .372 [.159; .585] |
| DG/CA4 | Left | 72 | .189 | .302 | .160 [-.081; .402] |
|  | Right | 84 | .016 | .050* | .255 [.048; .462] |
| SRLM | Left | 72 | .006 | .044* | .328 [.099; .558] |
|  | Right | 84 | .007 | .044* | .299 [.086; .513] |
| CA2/CA3 | Left | 72 | .072 | .143 | .216 [-0.20; .451] |
|  | Right | 84 | .196 | .302 | .142 [-.075; .358] |
| Subiculum | Left | 72 | .216 | .309 | .153 [-.091; .397] |
|  | Right | 84 | .195 | .302 | .149 [-.078; .377] |

Supplementary table 2: Summary of statistical tests for linear models of hippocampal subfield volumes with RAVLT recognition d’ for list A adjusted for age, sex, total intracranial volume and years of education.

| Hippocampal subfield | Hemisphere | DF | Uncorrected p-value | Adjusted p-value | Estimated partial β [95% CI] |
| --- | --- | --- | --- | --- | --- |
| CA1 | Left | 72 | .013 | .043* | .293 [.064; .522] |
|  | Right | 84 | .002 | .016* | .341 [.133; .550] |
| DG/CA4 | Left | 72 | .154 | .219 | .168 [-.064; .400] |
|  | Right | 84 | .049 | .099 | .204 [.001; .407] |
| SRLM | Left | 72 | .004 | .022* | .326 [.106; .547] |
|  | Right | 84 | .027 | .067 | .239 [.028; .449] |
| CA2/CA3 | Left | 72 | .191 | .239 | .152 [-.078; .381] |
|  | Right | 84 | .364 | .405 | .097 [-.114; .308] |
| Subiculum | Left | 72 | .062 | .103 | .221 [-.011; 453] |
|  | Right | 84 | .438 | .438 | .087 [-.135; .310] |

Supplementary table 3: Summary of statistical tests for linear models of hippocampal subfield volumes with AFLT delayed recall performance adjusted for age, sex, total intracranial volume and years of education.

| Hippocampal subfield | Hemisphere | DF | Uncorrected p-value | Adjusted p-value | Estimated partial β [95% CI] |
| --- | --- | --- | --- | --- | --- |
| CA1 | Left | 72 | .552 | .614 | .073 [-170; .316] |
|  | Right | 84 | .062 | .138 | .209 [-.011; .429] |
| DG/CA4 | Left | 72 | .361 | .451 | .110 [-.129; .349] |
|  | Right | 84 | .009 | .047* | .271 [.068; .474] |
| SRLM | Left | 72 | .318 | .425 | .119 [-.117; .356] |
|  | Right | 84 | .032 | .079 | .235 [.021; .449] |
| CA2/CA3 | Left | 72 | .553 | .614 | .071 [-.165; .307] |
|  | Right | 84 | .018 | .050* | .254 [.045; .462] |
| Subiculum | Left | 72 | .970 | .970 | .005 [-.238; .247] |
|  | Right | 84 | .667 | .702 | .049 [-.177; .276] |

Supplementary table 4: Summary of statistical tests for linear models of hippocampal subfield volumes with AFLT recognition d’ for list A adjusted for age, sex, total intracranial volume and years of education.

| Hippocampal subfield | Hemisphere | DF | Uncorrected p-value | Adjusted p-value | Estimated partial β [95% CI] |
| --- | --- | --- | --- | --- | --- |
| CA1 | Left | 72 | .537 | .775 | -.074 [-.313; .165] |
|  | Right | 83 | .209 | .696 | .140 [-.081; .365] |
| DG/CA4 | Left | 72 | .129 | .644 | -.177 [-.402; .052] |
|  | Right | 83 | .617 | .775 | .052 [-.155; .259] |
| SRLM | Left | 72 | .320 | .775 | -.116 [-.345; .114] |
|  | Right | 83 | .502 | .775 | .074 [-.145; .294] |
| CA2/CA3 | Left | 72 | .071 | .644 | -.208 [-.428; .018] |
|  | Right | 83 | .965 | .965 | -.004 [-.215; .205] |
| Subiculum | Left | 72 | .697 | .775 | -.046 [-.285; .191] |
|  | Right | 83 | .673 | .775 | .048 [-.178; .275] |

Supplementary table 5: Summary of statistical tests for linear models of hippocampal subfield volumes with AFLT recognition d’ for list B adjusted for age, sex, total intracranial volume and years of education.

| Hippocampal subfield | Hemisphere | DF | Uncorrected p-value | Adjusted p-value | Estimated partial β [95% CI] |
| --- | --- | --- | --- | --- | --- |
| CA1 | Left | 65 | .729 | .911 | .045 [-.209; .298] |
|  | Right | 77 | .367 | .911 | .108 [-.130; .348] |
| DG/CA4 | Left | 65 | .494 | .911 | .086 [-.165; .338] |
|  | Right | 77 | .642 | .911 | .052 [-.169; .272] |
| SRLM | Left | 65 | .949 | .949 | .008 [-.235; .251] |
|  | Right | 77 | .519 | .911 | .075 [-.158; .310] |
| CA2/CA3 | Left | 65 | .640 | .911 | -.058 [-.305; .189] |
|  | Right | 77 | .625 | .911 | -.057 [-.274; .166] |
| Subiculum | Left | 65 | .223 | .911 | -.153 [-.405; .096] |
|  | Right | 77 | .854 | .949 | .022 [-.213; .257] |

Supplementary table 6: Summary of statistical tests for ANCOVAs of hippocampal subfield volumes with amyloid positivity adjusted for age, sex, total intracranial volume, years of education and APOE ε4 carriership.

| Hippocampal subfield | Hemisphere | F-statistic | DF | Uncorrected p-value | Adjusted p-value | Adjusted SMD [95% CI] |
| --- | --- | --- | --- | --- | --- | --- |
| CA1 | Left | 1.35 | 71 | .180 | .225 | .336 [-.159; .832] |
|  | Right | 2.045 | 83 | .044 | .086 | .479 [.013; .945] |
| DG/CA4 | Left | 2.08 | 71 | .041 | .086 | .482 [.020; .944] |
|  | Right | 2.11 | 83 | .038 | .086 | .446 [.026; .866] |
| SRLM | Left | 1.76 | 71 | .083 | .128 | .410 [-.055; .876] |
|  | Right | 2.01 | 83 | .047 | .086 | .449 [.005; .892] |
| CA2/CA3 | Left | 1.55 | 71 | .125 | .179 | .369 [-.105; .842] |
|  | Right | 1.22 | 83 | .226 | .266 | .271 [-.172; .715] |
| Subiculum | Left | 0.67 | 71 | .545 | .574 | .150 [-.642; .342] |
|  | Right | 1.11 | 83 | .270 | .300 | .264 [-.209; .738] |

Supplementary table 7: Summary of statistical tests for ANCOVAs of hippocampal subfield volumes with tau positivity adjusted for age, sex, total intracranial volume, years of education and APOE ε4 carriership.

| Hippocampal subfield | Hemisphere | F- statistic | DF | Uncorrected p-value | Adjusted p-value | Adjusted SMD [95% CI] |
| --- | --- | --- | --- | --- | --- | --- |
| CA1 | Left | 2.06 | 71 | .043 | .086 | .491 [.015; .966] |
|  | Right | 2.51 | 83 | .014 | .056 | .585 [.122; 1.049] |
| DG/CA4 | Left | 4.20 | 71 | .000 | .002* | .876 [.460; 1.292] |
|  | Right | 2.63 | 83 | .010 | .050* | .552 [.135; .970] |
| SRLM | Left | 2.95 | 71 | .004 | .029* | .649 [.210; 1.087] |
|  | Right | 2.42 | 83 | .018 | .059 | .538 [.096; .981] |
| CA2/CA3 | Left | 3.10 | 71 | .002 | .028* | .686 [.245; 1.127] |
|  | Right | 1.77 | 83 | .080 | .128 | .394 [-.048; .837] |
| Subiculum | Left | 0.26 | 71 | .797 | .797 | .062 [-.419; .544] |
|  | Right | 1.37 | 83 | .174 | .225 | .328 [-.147; .802] |


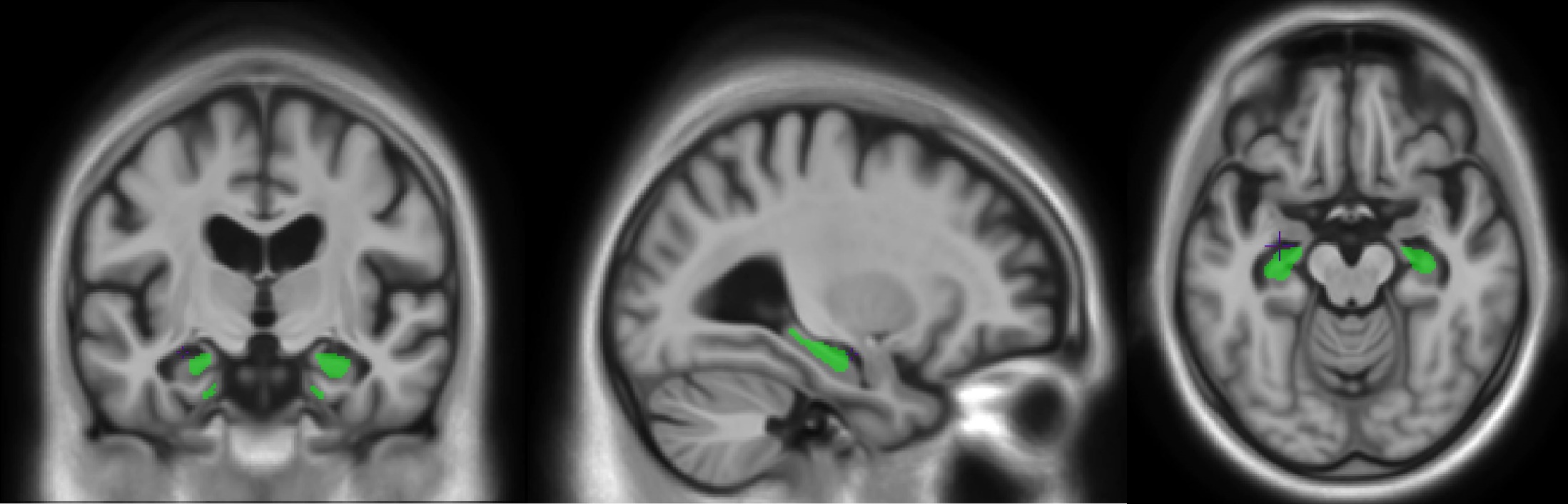


**Supplementary figure 1: Braak stage II region (green) over the ADMI template image (greyscale).** This mask was used to extract [18F]MK-6240 uptake to classify subjects as either tau positive or negative.

**
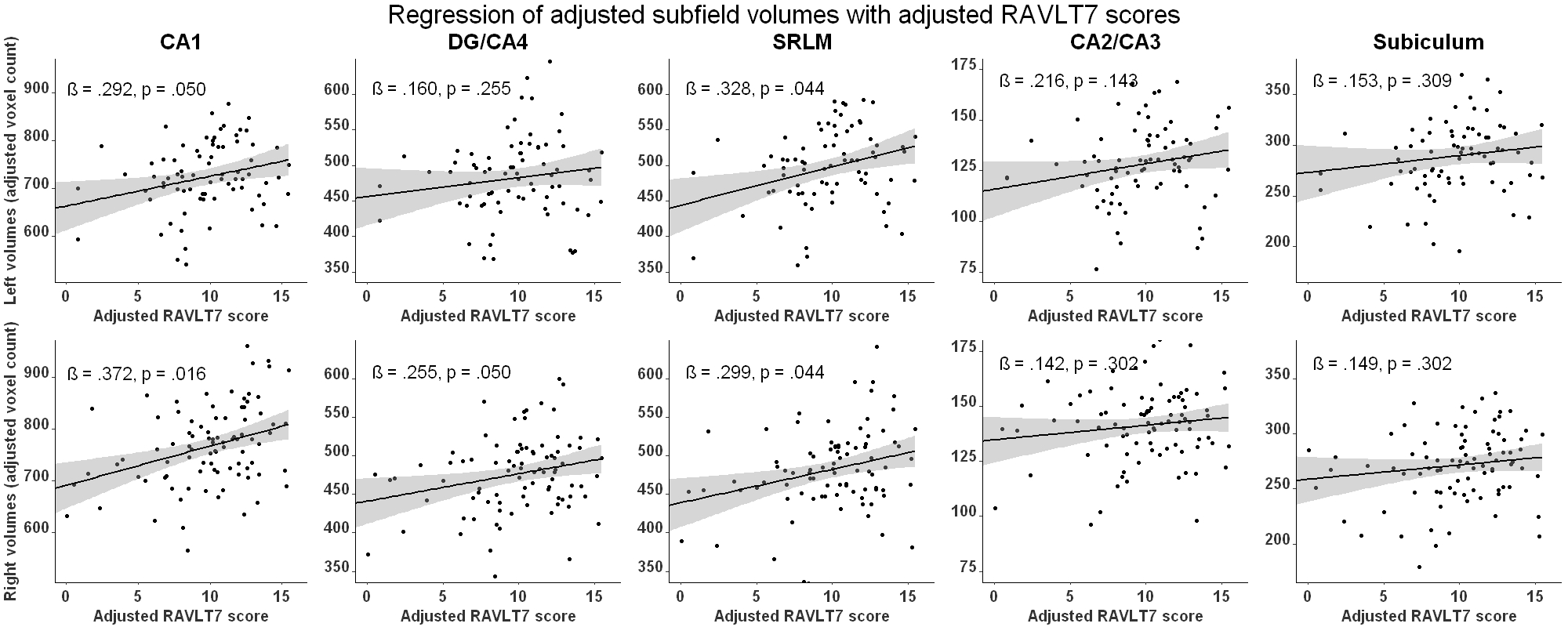
**

**Supplementary figure 2: Representation of semi-partial regressions between hippocampal subfield volumes and delayed verbal free recall performance (RAVLT7).** Data were adjusted for age, sex, years of education and total intracranial volume. Left subfield N = 77, right subfield N = 89. Displayed p-values are FDR-corrected. β = standardized semi-partial regression coefficient; CA = Cornu ammonis; DG = Dentate gyrus; SRLM = Strata radiatum, lacunosum and moleculare.


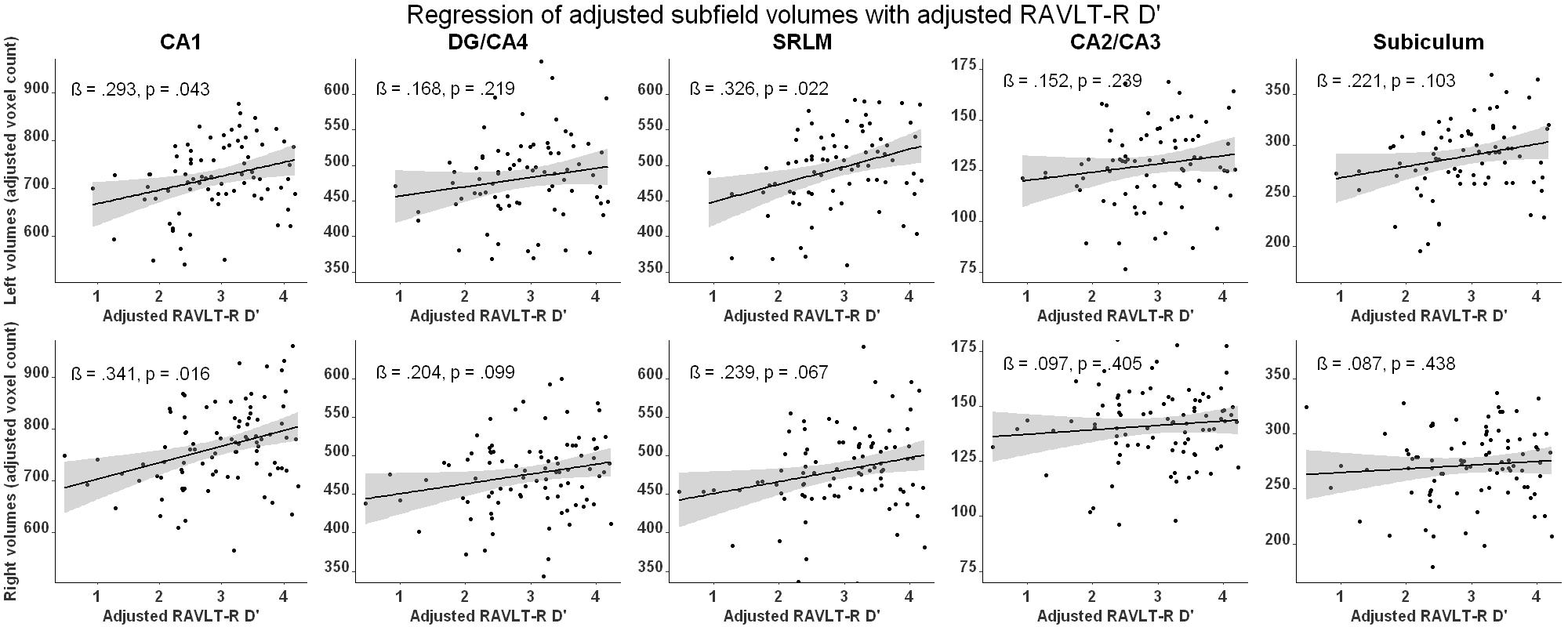


**Supplementary figure 3: Representation of semi-partial regressions between hippocampal subfield volumes and delayed verbal recognition (RAVLT-R) discrimination index (d’).** Data were adjusted for age, sex, years of education and total intracranial volume. Left subfield N = 77, right subfield N = 89. Displayed p-values are FDR-corrected. β = standardized semi-partial regression coefficient; CA = Cornu ammonis; DG = Dentate gyrus; SRLM = Strata radiatum, lacunosum and moleculare.


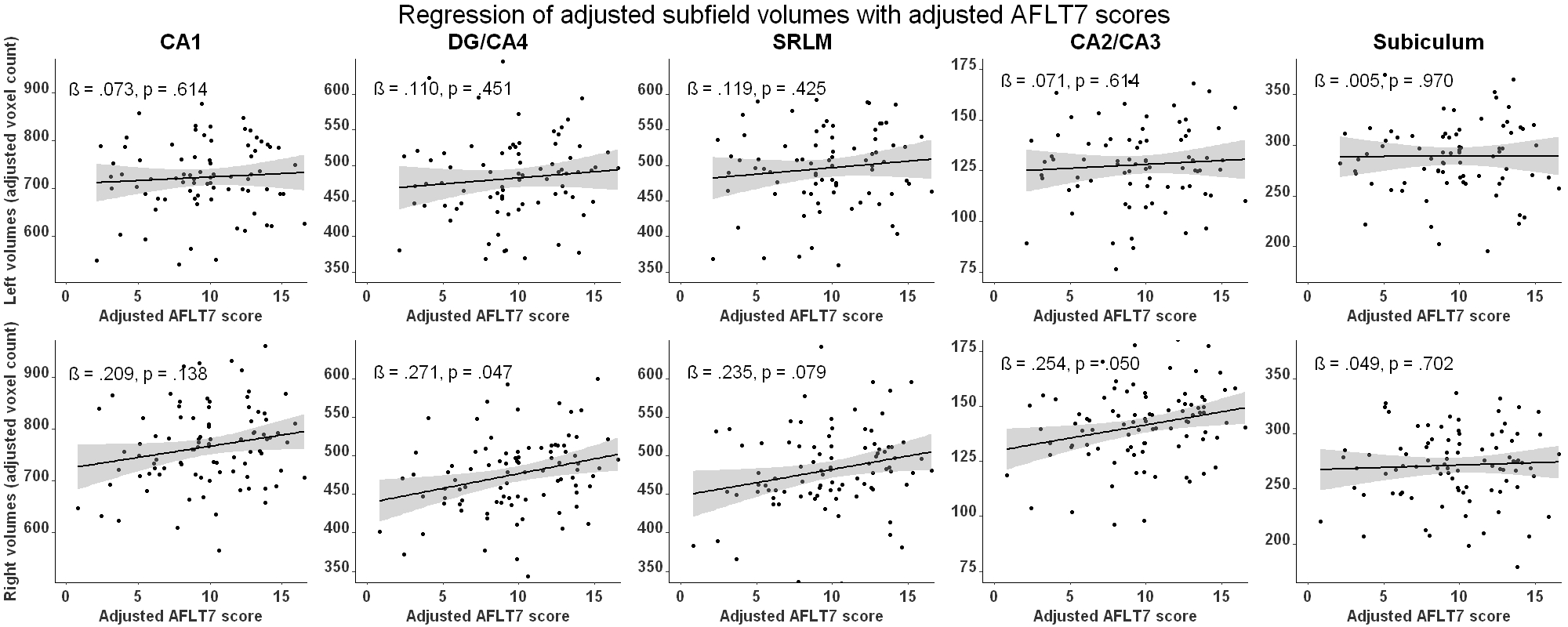


**Supplementary figure 4: Representation of semi-partial regressions between hippocampal subfield volumes and delayed visual free recall performance (AFLT7).** Data were adjusted for age, sex, years of education and total intracranial volume. Left subfield N = 77, right subfield N = 89. Displayed p-values are FDR-corrected. β = standardized semi-partial regression coefficient; CA = Cornu ammonis; DG = Dentate gyrus; SRLM = Strata radiatum, lacunosum and moleculare.


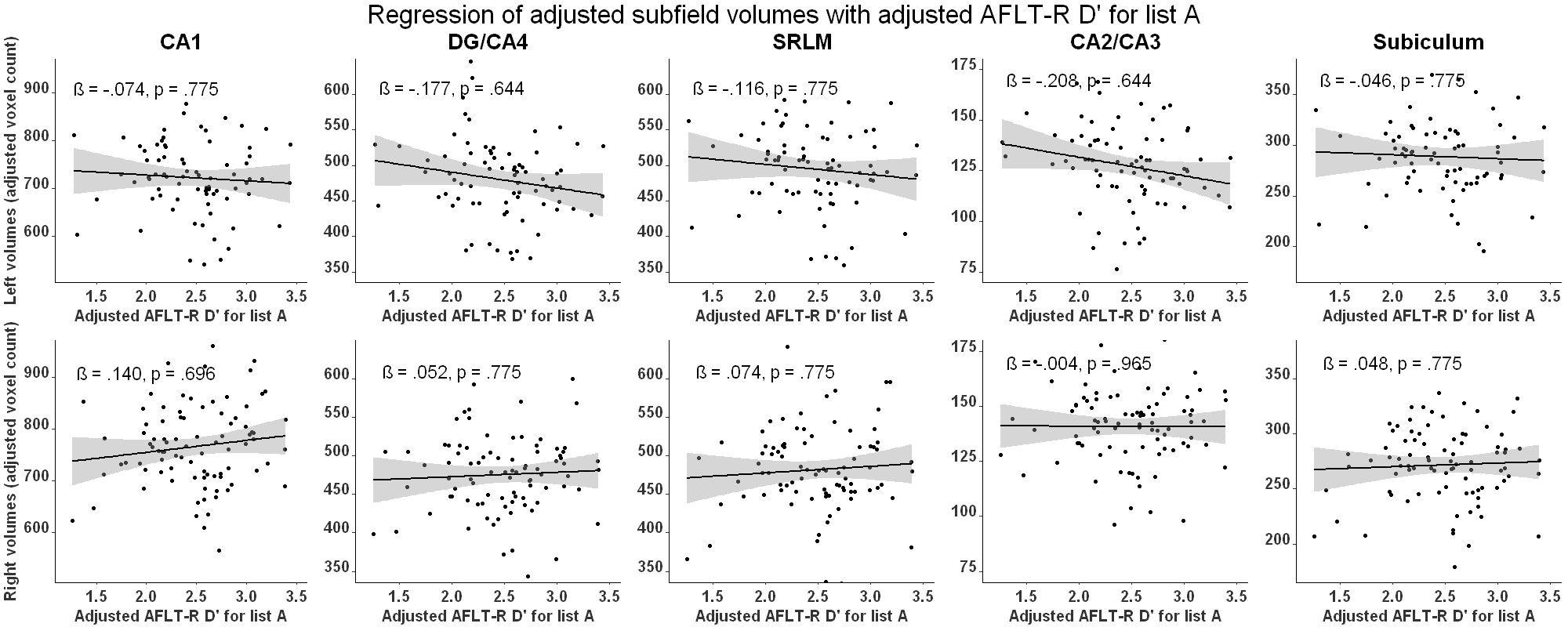


**Supplementary figure 5: Representation of semi-partial regressions between hippocampal subfield volumes and delayed visual recognition (AFLT-R) discrimination index for series A (d’).** Data were adjusted for age, sex, years of education and total intracranial volume. Left subfield N = 77, right subfield N = 89. Displayed p-values are FDR-corrected. β = standardized semi-partial regression coefficient; CA = Cornu ammonis; DG = Dentate gyrus; SRLM = Strata radiatum, lacunosum and moleculare.


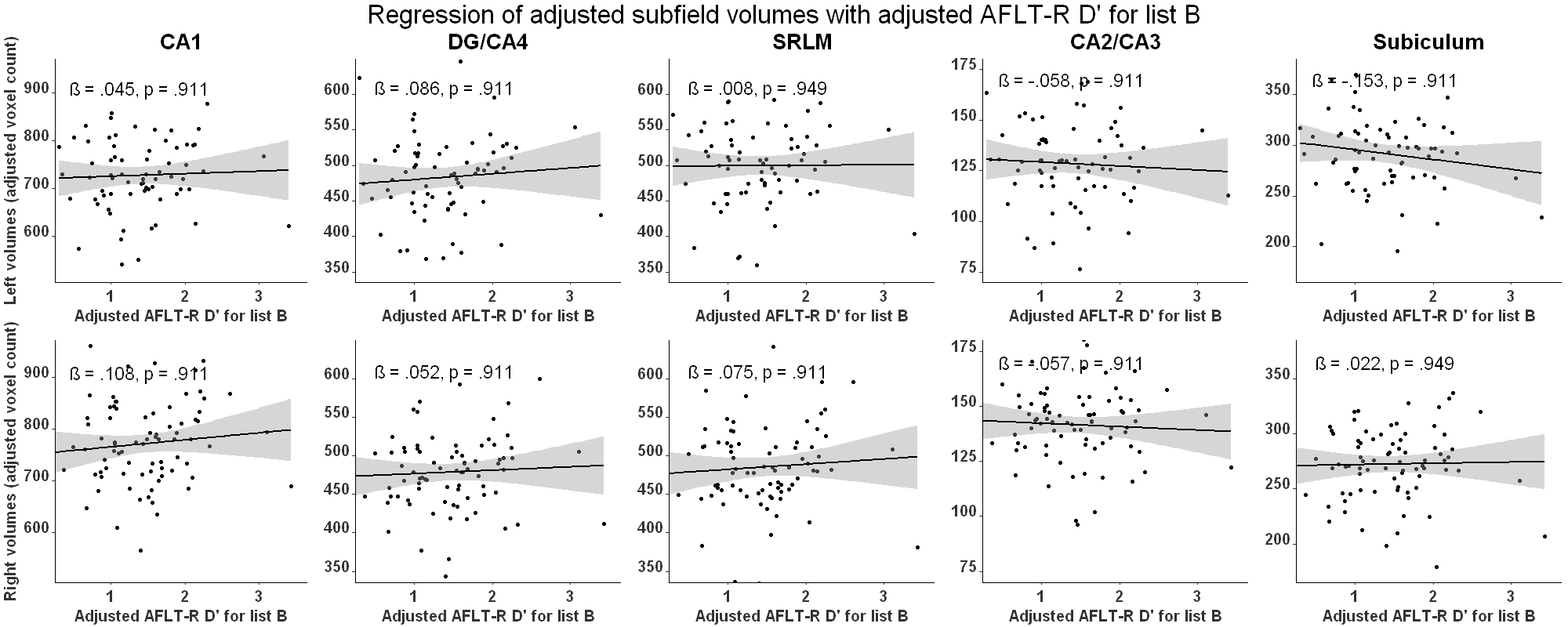


**Supplementary figure 6: Representation of semi-partial regressions between hippocampal subfield volumes and delayed visual recognition (AFLT-R) discrimination index for series B (d’).** Data were adjusted for age, sex, years of education and total intracranial volume. Left subfield N = 77, right subfield N = 89. Displayed p-values are FDR-corrected. β = standardized semi-partial regression coefficient; CA = Cornu ammonis; DG = Dentate gyrus; SRLM = Strata radiatum, lacunosum and moleculare.


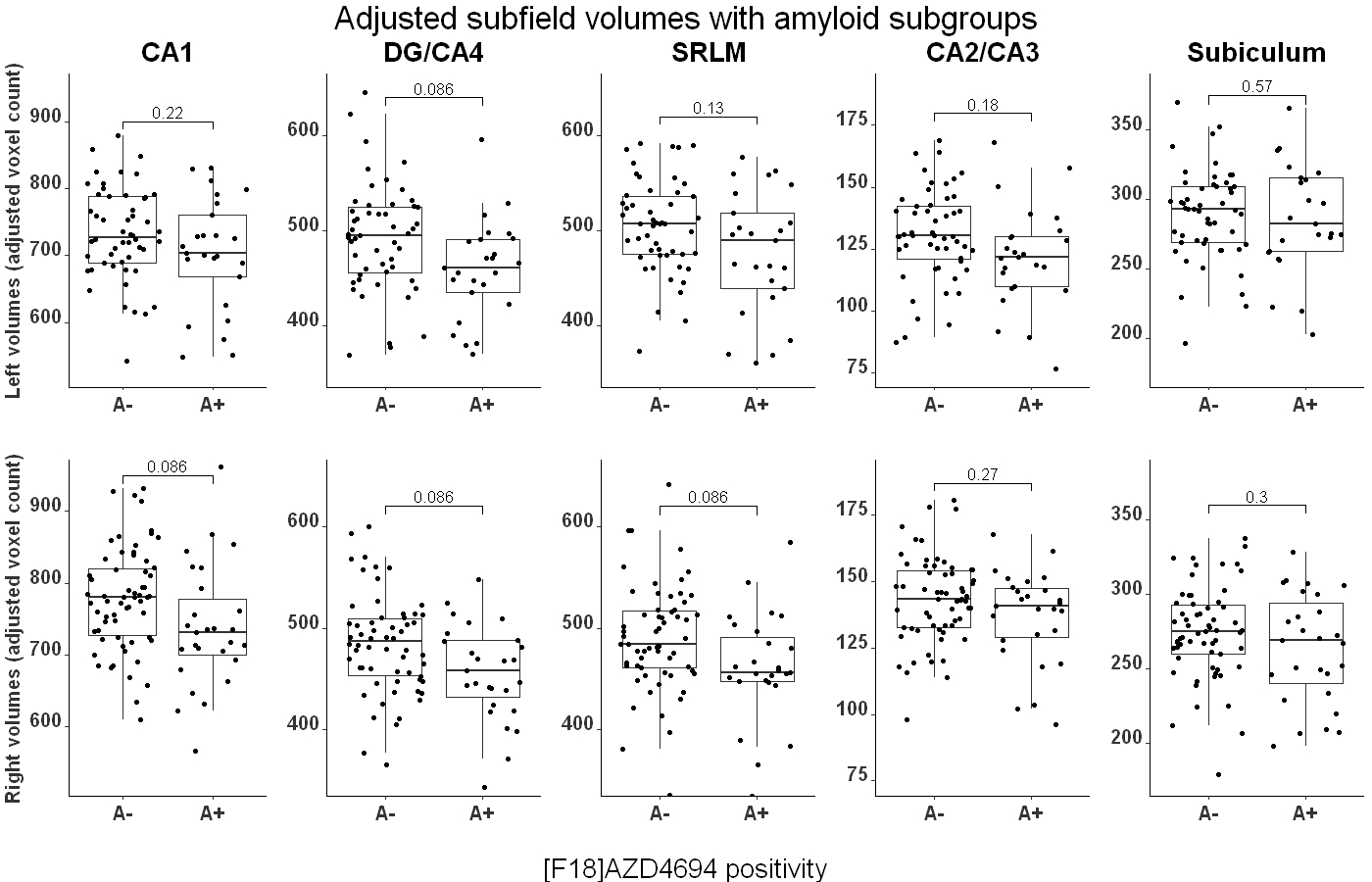


**Supplementary figure 7: Hippocampal subfield volumes for amyloid-positive (A+) and amyloid-negative (A-) participants.** Data points are adjusted for age, sex, total intracranial volume, years of education and APOE ε4 carrier status. Left subfield N = 77, right subfield N = 89. Displayed p-values represent results from FDR-corrected ANCOVAs. CA = Cornu ammonis; DG = Dentate gyrus; SRLM = Strata radiatum, lacunosum and moleculare.


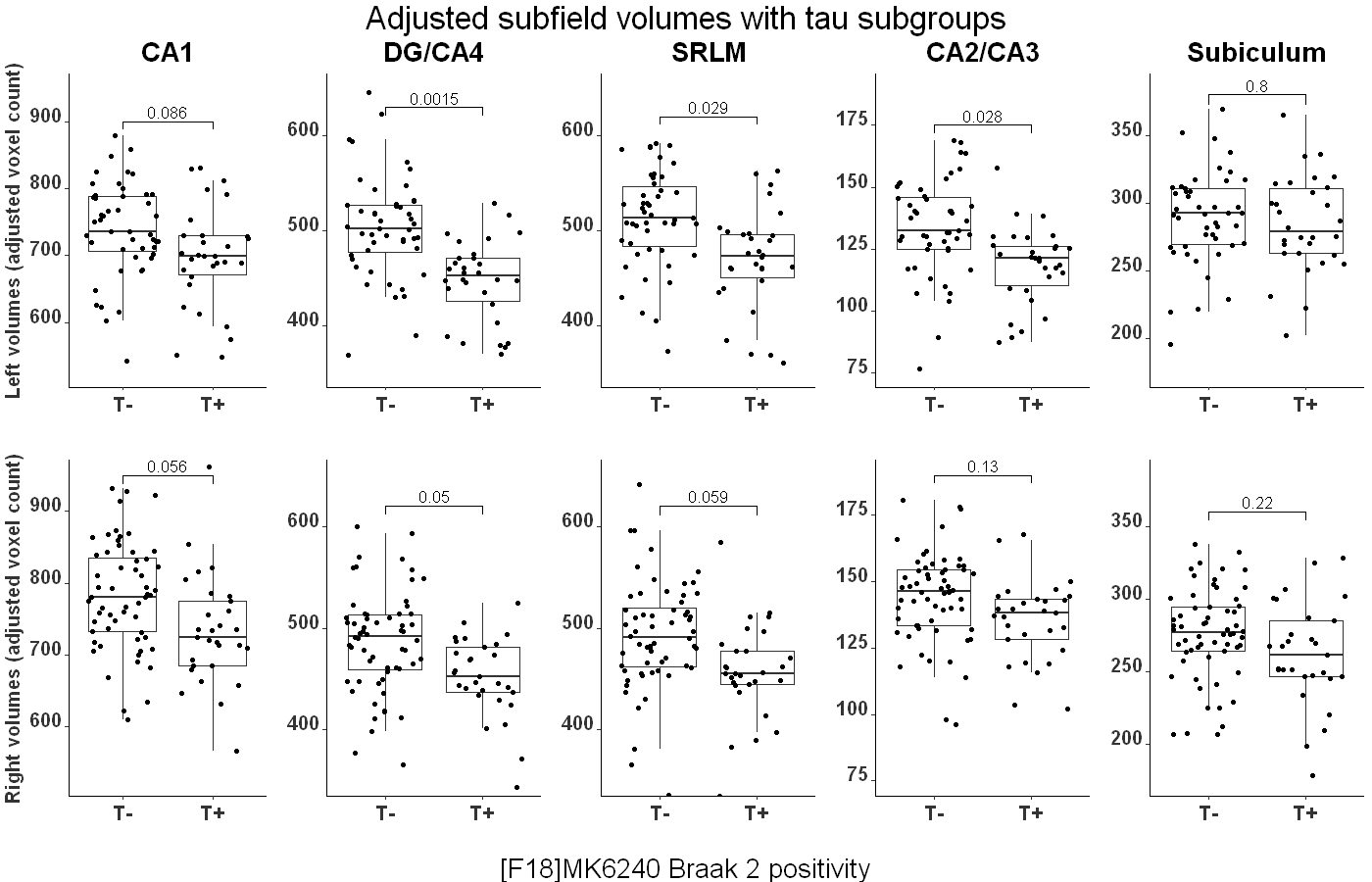


**Supplementary figure 8: Hippocampal subfield volumes for tau-positive (T+) and tau-negative (T-) participants.** Data points are adjusted for age, sex, total intracranial volume, years of education and APOE ε4 carrier status. Left subfield N = 77, right subfield N = 89. Displayed p-values represent results from FDR-corrected ANCOVAs. CA = Cornu ammonis; DG = Dentate gyrus; SRLM = Strata radiatum, lacunosum and moleculare.


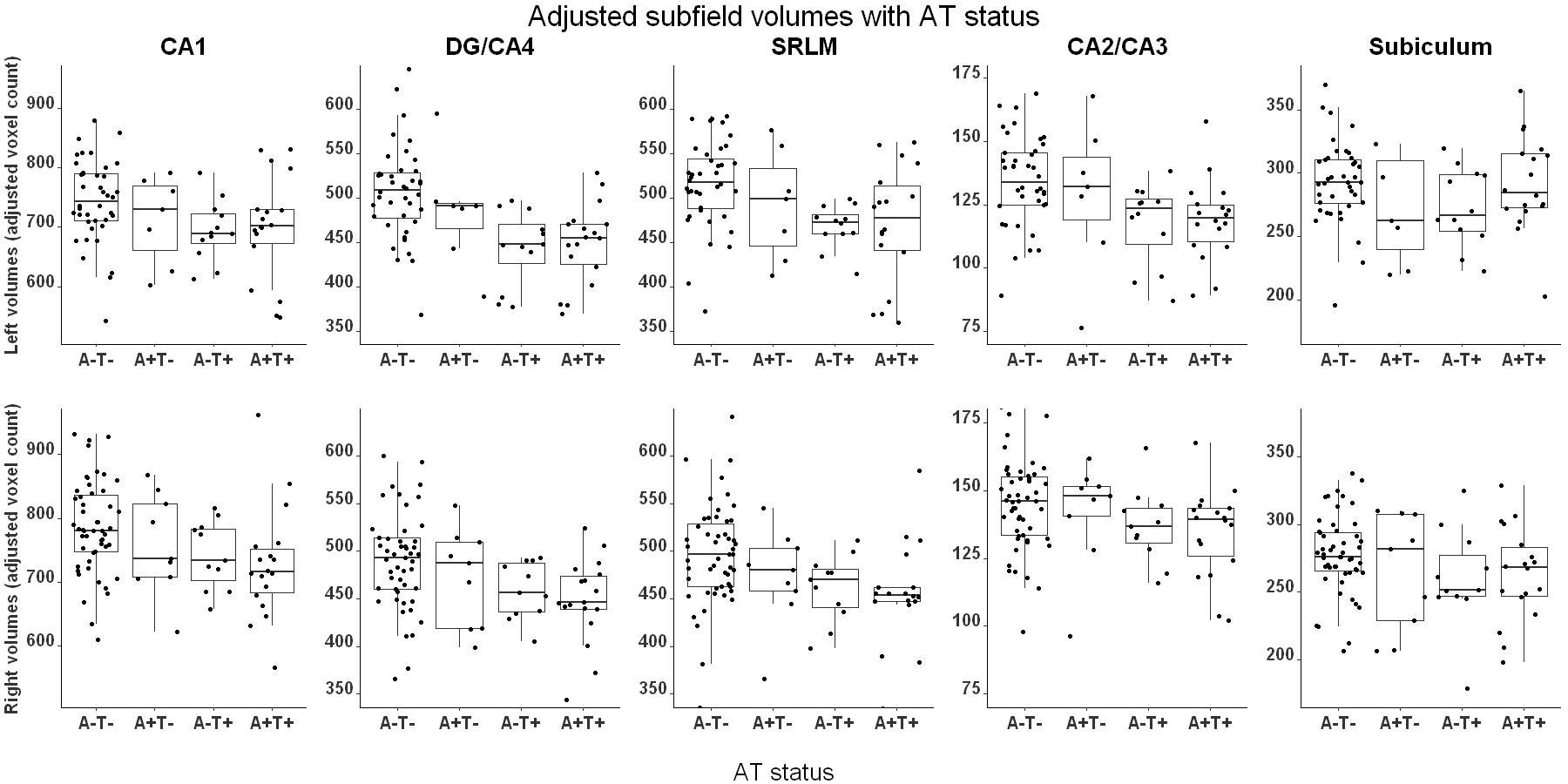


**Supplementary figure 9: Hippocampal subfield volumes for amyloid and tau negative (A-T-), Amyloid positive and tau negative (A+T-), amyloid negative and tau positive (A-T+) and amyloid and tau positive (A+T+) participants.** Data points are adjusted for age, sex, total intracranial volume, years of education and APOE ε4 carrier status. Left subfield N = 77, right subfield N = 89. CA = Cornu ammonis; DG = Dentate gyrus; SRLM = Strata radiatum, lacunosum and moleculare.
